# Supplementary material for: Impact of expanding smoke-free policies beyond enclosed public places and workplaces on children’s tobacco smoke exposure and respiratory health: protocol for a systematic review and meta-analysis
Source: BMJ Open. 2020 Oct 19;10(10):e038234. doi: 10.1136/bmjopen-2020-038234 (PMC7577335; doi:10.1136/bmjopen-2020-038234)
Supplement: Supplementary data [file bmjopen-2020-038234supp001.pdf]

Appendix:

Search terms used for Embase.com database

('smoking regulation'/exp OR 'smoking ban'/de OR (smokefree OR ((smoking OR smoke OR tobacco) NEAR/3 (regulation* OR government* OR law OR laws OR policy OR policies OR ban OR bans OR banned OR free OR restrict* OR act OR acts))):ab,ti) AND (child/exp OR pediatrics/exp OR childhood/exp OR 'child welfare'/de OR 'child development'/de OR 'child health'/de OR 'child health care'/exp OR 'child care'/exp OR 'childhood disease'/exp OR 'pediatric ward'/de OR 'pediatric hospital'/de OR (infan* OR child* OR kid OR kids OR toddler* OR boy* OR girl* OR minors OR underag* OR (under NEXT/1 (age* OR aging)) OR kindergar* OR prepubescen* OR prepubert* OR pediatric* OR paediatric* OR school* OR preschool* OR (age NEAR/3 analy*)):ab,ti) AND ('asthma'/exp OR 'wheezing'/exp OR 'respiratory tract infection'/exp OR 'coughing'/exp OR 'otitis media'/exp OR 'exposure'/de OR 'self report'/exp OR cotinine/exp OR nicotine/exp OR 'saliva analysis'/exp OR 'urinalysis'/exp OR 'electronic device'/de OR 'wearable device'/de OR 'wearable sensor'/de OR 'hair analysis'/exp OR 'skin analysis device'/de OR 'passive smoking'/de OR 'environmental exposure'/de OR 'lung function test'/exp OR 'lung volume'/exp OR (asthma* OR wheezing OR ((respirator* OR lung OR pulmonar* OR expirat*) NEAR/3 (infection* OR function* OR volume*)) OR pneumonia OR bronchiolitis* OR cough* OR (otitis NEAR/3 media) OR exposure* OR environment* OR self-report* OR parent*-report* OR carer*-report* OR nicotine OR cotinin* OR second-hand* OR third-hand* OR secondhand* OR thirdhand* OR (passive NEAR/3 smoking) OR ((urine* OR saliva* OR hair OR hand* OR skin) NEAR/3 analy*) OR urinalys* OR ((electronic OR wearable) NEAR/3 (device* OR sensor*)) OR spirometr* OR fev OR fev1 OR fvc OR (forced NEAR/3 vital NEAR/3 capacit*) OR Tiffeneau):ab,ti) NOT ([Conference Abstract]/lim OR [editorial]/lim OR [letter]/lim)

Bias assessment criteria (confounders and co-interventions)

ROBINS-I requires to pre-specify certain risk-of-bias criteria that are relevant for the given study. For assessing the risk of bias due to confounding, we consider the following potential confounders relevant: age, gender, socio-economic status (SES) and/or ethnicity, and the pre-implementation temporal trend in the given outcome variable. We will score results as having a low risk of bias due to confounding if it controls for all of these variables, moderate risk of bias if not all confounders are controlled for but the pre-implementation trend is, high risk of bias if the pre- implementation trend is not controlled for but some of the other confounders are, and critical risk of bias if none of the listed confounders are controlled for. Other potential contextual confounders specifically for the child health outcomes are air pollution, quality of the health care system, access to health care facilities, and the vaccination programme in the country. Therefore, additionally to the criteria noted above, studies will also be considered to have a critical risk of bias due to confounding if contextual confounders were unaccounted for despite these being reported to be an issue in the study.

For assessing bias due to co-interventions (i.e. part of ‘bias due to deviations from intended interventions’), the following co-interventions are considered: policies aimed at reducing ambient air pollution, introduction of relevant health-care reforms and/or public health programmes, introduction of other tobacco control policies, change in vaccination programmes, and wars/epidemics/economic crises which influenced the health of children. We will approximate the percentage of the observation units affected by any of these uncontrolled co-interventions. When this percentage is below 10% a result will be considered to have a low risk of bias, when it ranges between 10% and 20% moderate risk of bias, between 20% and 40% high risk of bias, and above 40% it will be considered serious risk of bias.

Radó MK, *et al. BMJ Open* 2020; 10:e038234. doi: 10.1136/bmjopen-2020-038234

Appendix Table 1. Bias due to confounding and due to deviations from intended interventions

Level of bias	Bias due to confounding	Bias due to deviations from intended interventions
**Low risk of bias**	(I) The pre-implementation temporal trends in the outcomes are controlled<br>(II) Individual characteristics are controlled<br>(III) (For health outcomes): Contextual characteristics are controlled if reported to be an issue in the study	The percentage of the units affected by any of the co-interventions is below 10%
**Moderate risk of bias**	(I) The pre-implementation temporal trends in the outcomes are controlled<br>(II) Individual characteristics are not all controlled<br>(III) (For health outcome): Contextual characteristics are controlled if reported to be an issue in the study	The percentage of the units affected by any of co-interventions is between 10% and 20%
**Serious risk of bias**	(I) The pre-implementation temporal trends in the outcomes are not controlled<br>(II) Some of the individual characteristics are controlled<br>(III) (For health outcome): Contextual characteristics are controlled if reported to be an issue in the study	The percentage of the units affected by any of co-interventions is between 20% and 40%
**Critical risk of bias**	(I) The pre-implementation temporal trends in the outcomes are not controlled<br>(II) Some of individual characteristics are controlled<br>**OR**<br>(III) (FOR health outcome): Contextual characteristics are not controlled while being reported to be an issue in the study	The percentage of the units affected by any of co-interventions is above 40%
**No information**	No information on the controlled confounding variables	No information on the controlled co-interventions

Radó MK, *et al. BMJ Open* 2020; 10:e038234. doi: 10.1136/bmjopen-2020-038234
