# Supplementary material for: Impact of expanding smoke-free policies beyond enclosed public places and workplaces on children’s tobacco smoke exposure and respiratory health: protocol for a systematic review and meta-analysis
Source: BMJ Open. 2020 Oct 19;10(10):e038234. doi: 10.1136/bmjopen-2020-038234 (PMC7577335; doi:10.1136/bmjopen-2020-038234)
Supplement: Author's manuscript [file bmjopen-2020-038234.draft_revisions.pdf]

BMJ Open is committed to open peer review. As part of this commitment we make the peer review history of every article we publish publicly available.

When an article is published we post the peer reviewers' comments and the authors' responses online. We also post the versions of the paper that were used during peer review. These are the versions that the peer review comments apply to.

The versions of the paper that follow are the versions that were submitted during the peer review process. They are not the versions of record or the final published versions. They should not be cited or distributed as the published version of this manuscript.

BMJ Open is an open access journal and the full, final, typeset and author-corrected version of record of the manuscript is available on our site with no access controls, subscription charges or pay-per-view fees (http://bmjopen.bmj.com).

If you have any questions on BMJ Open's open peer review process please email info.bmjopen@bmj.com

BMJ Open

Impact of expanding smoke-free policies beyond enclosed public places on children's tobacco smoke exposure and respiratory health: protocol for a systematic review

Journal:	*BMJ Open*
Manuscript ID	bmjopen-2020-038234
Article Type:	Protocol
Date Submitted by the Author:	04-Mar-2020

Complete List of Authors:	Rado, Márta; Erasmus University Medical Centre, Department of Paediatrics; Erasmus University Medical Centre , Department of Public Health<br>Mölenberg, Famke; Erasmus Medical Center, Public Health<br>Sheikh, Aziz; University of Edinburgh, Division of Community Health Sciences<br>Millett, Christopher; Imperial College, Primary Care and Social Medicine<br>Bramer, Wichor; Erasmus MC, Medical Library<br>Burdorf, Alex; Erasmus MC, Department of Public Health<br>Van Lenthe, Frank; Erasmus University Medical Centre, Department of Public Health<br>Been, Jasper; Erasmus University Medical Centre–Sophia Children's Hospital, Paediatrics

Keywords:	Health policy < HEALTH SERVICES ADMINISTRATION & MANAGEMENT, Child protection < PAEDIATRICS, Community child health < PAEDIATRICS, PUBLIC HEALTH, Respiratory infections < THORACIC MEDICINE

SCHOLARONE™
Manuscripts

I, the Submitting Author has the right to grant and does grant on behalf of all authors of the Work (as defined in the below author licence), an exclusive licence and/or a non-exclusive licence for contributions from authors who are: i) UK Crown employees; ii) where BMJ has agreed a CC-BY licence shall apply, and/or iii) in accordance with the terms applicable for US Federal Government officers or employees acting as part of their official duties; on a worldwide, perpetual, irrevocable, royalty-free basis to BMJ Publishing Group Ltd ("BMJ") its licensees and where the relevant Journal is co-owned by BMJ to the co-owners of the Journal, to publish the Work in this journal and any other BMJ products and to exploit all rights, as set out in our licence.

The Submitting Author accepts and understands that any supply made under these terms is made by BMJ to the Submitting Author unless you are acting as an employee on behalf of your employer or a postgraduate student of an affiliated institution which is paying any applicable article publishing charge ("APC") for Open Access articles. Where the Submitting Author wishes to make the Work available on an Open Access basis (and intends to pay the relevant APC), the terms of reuse of such Open Access shall be governed by a Creative Commons licence – details of these licences and which Creative Commons licence will apply to this Work are set out in our licence referred to above.

Other than as permitted in any relevant BMJ Author's Self Archiving Policies, I confirm this Work has not been accepted for publication elsewhere, is not being considered for publication elsewhere and does not duplicate material already published. I confirm all authors consent to publication of this Work and authorise the granting of this licence.

For peer review only

Impact of expanding smoke-free policies beyond enclosed public places on children's tobacco smoke exposure and respiratory health: protocol for a systematic review

Authors: Marta K. Rado [ab], Famke J.M. Mölenberg [b], Aziz Sheikh [cd], Christopher Millett [e], Wichor M. Bramer [f], Alex Burdorf [b], Frank J. van Lenthe [b], Jasper V. Been [abc] *

[a] Division of Neonatology, Department of Paediatrics, Erasmus MC — Sophia Children's Hospital, University Medical Centre Rotterdam, Rotterdam, Netherlands

[b] Department of Public Health, Erasmus MC, University Medical Centre Rotterdam, Rotterdam, Netherlands

[c] Centre of Medical Informatics, Usher Institute, The University of Edinburgh, Edinburgh, UK

[d] Division of General Internal Medicine and Primary Care, Brigham and Women's Hospital/Harvard Medical School, Boston, MA, USA

[e] Public Health Policy Evaluation Unit, School of Public Health, Imperial College London, London, UK

[f] Medical Library, Erasmus MC, University Medical Centre Rotterdam, Rotterdam, Netherlands

*To whom correspondence should be addressed:

Dr Jasper V. Been, MD MPH PhD

Erasmus MC— Sophia Children's Hospital,

Rotterdam, Netherlands PO Box 2060, 3000 CB Rotterdam

Phone: +31-10-7037048

Email: j.been@erasmusmc.nl

Keywords: smoke free policy, child health, tobacco smoke, respiratory health, meta-analysis

Word count: 4245

For peer review only - http://bmjopen.bmj.com/site/about/guidelines.xhtml

ABSTRACT

Introduction: Tobacco smoke exposure (TSE) has considerable adverse respiratory health impact among children. Smoke-free policies covering enclosed public places are known to reduce child TSE and benefit child health. An increasing number of jurisdictions are now expanding smoke-free policies to also cover outdoor areas and/or (semi-) private spaces (indoor and/or outdoor). We aim to systematically review the evidence on the impact of these 'novel smoke-free policies' on children's TSE and respiratory health.

Methods and analysis: 13 electronic databases will be searched by two independent reviewers for eligible studies. We will consult experts from the field and hand-search references and citations to identify additional published and unpublished studies. Study designs recommended by the Cochrane Effective Practice and Organisation of Care (EPOC) group are eligible, without restrictions on the observational period, publication date, or language. Our primary outcomes are: self- or parental-reported TSE in places covered by the policy; unplanned hospital attendance for wheezing/asthma; and unplanned hospital attendance for respiratory infections. We will assess risk of bias of individual studies following the EPOC or ROBINS-I tool, as appropriate. We will conduct separate random-effects meta-analyses for smoke-free policies covering (1) indoor private places, (2) indoor semi-private places, (3) outdoor (semi)private places, and (4) outdoor public places. We will assess whether the policies were associated with changes in TSE in other locations (e.g. displacement). Subgroup analyses will be conducted based on country income classification (i.e. high, low or middle-income) and by socioeconomic status (SES). Sensitivity analyses will be undertaken via broadening our study design eligibility criteria (i.e. including non-EPOC designs) or via excluding studies with a high risk of bias. This review will inform policymakers regarding the implementation of extended smoke-free policies to safeguard children's health.

Ethics and dissemination: Ethical approval is not required. Findings will be disseminated to the academicsand the general public.

Strengths and limitations of this study

- This systematic review will be the first to comprehensively synthesise the evidence regarding the impact of 'novel smoke-free policies' (i.e. those covering (1) indoor private places, (2) indoor semi-private places, (3) outdoor (semi)private places, and (4) outdoor public places).
- This protocol has been designed in line with the Preferred Reporting Items for Systematic Reviews and Meta-Analyses for Protocols (PRISMA-P) guidelines and guidelines of the Cochrane Effective Practice and Organisation of Care (EPOC)
- A possible limitation is that included studies might be heterogeneous in the policy, study design and data collection methods which might limit our ability to synthesise the results using meta-analysis
- The value of this systematic review depends on the quality and availability of the evidence on the topic
- This work will be instrumental in informing effective policymaking regarding implementing novel smoke-free policies to protect children from the adverse effects of tobacco smoke.

For peer review only - http://bmjopen.bmj.com/site/about/guidelines.xhtml

1. INTRODUCTION

Every second child worldwide is regularly exposed to tobacco smoke, leading to adverse child health outcomes. Tobacco smoke exposure (TSE) includes both second-hand smoke (SHS) and third-hand smoke (THS) exposure. SHS refers to the inhalation of emitted smoke, while THS exposure refers to the uptake of tobacco smoke residuals from polluted surfaces after someone has finished smoking. TSE is estimated to cause 166,000 child deaths annually associated with an increased risk of respiratory tract infections (RTI), wheezing, and asthma.[1 2]

Safeguarding of child health provides justification to advocate for strong tobacco control measures.[3] Previous systematic reviews found that smoke-free policies in enclosed public places are successful in reducing adverse child health outcomes, including hospital attendance for asthma exacerbations (–9.8% [95% confidence interval (CI) –16.6 to –3.0]) and hospital attendance for lower RTIs (–18.5% [95% CI –32.8 to –4.2]).[2] These health impacts are likely mediated via a reduction in SHS and potentially also in THS.[1] Indeed, individual studies have demonstrated that smoke-free policies covering enclosed public areas are successful in decreasing child TSE in public places and even in private places, such as cars or homes, most likely via changes in social norms.[4-8]

As a result, smoke-free legislation is increasingly recognised as important policy tool to protect children from the adverse health effects of TSE (e.g. incorporated in Sustainable Development Goal 3.2, 3.4, 3.9, and 3.A that aims to improve health and well-being).[3] Recently, policies to provide additional protection of children to TSE beyond enclosed public places have been implemented in a number of places, both at a national and a sub-national level. These include expansion to cover outdoor areas frequented by children such as school grounds, playgrounds and parks.[9] Also, some countries and subnational regions have now implemented policies prohibiting smoking in semi-private spaces such as shared housing or private spaces such as cars.[10 11]

The positive effects of smoke-free policies covering enclosed public places on child health are now well established.[2 12] A systematic synthesis of the evidence on the effectiveness of smoke-free policies covering outdoor and/or (semi)private spaces to reduce children's TSE and benefit their health, is however currently lacking.[2 12] It is important to note that effectiveness of smoke-free policies covering indoor public places cannot be easily extrapolated to those covering outdoor or private spaces, due to various reasons (e.g. dilution of TSE in outdoor spaces, enforcement issues). Furthermore it is unknow whether smoke-free policies in these places may affect TSE exposure in other places either negatively (i.e. via displacement of smoking) or positively (i.e. via norm spreading).[11]

With an increasing number of institutions and governments now implementing smoke-free policies covering areas other than enclosed public spaces, optimal understanding of the effectiveness of such policies in protecting children is essential. To establish this evidence base, we will undertake a comprehensive systematic review of available studies assessing the impact of policies covering outdoor areas or/and (semi)private places (whether indoor or outdoor) on children's TSE and respiratory health. We believe this work will be instrumental to informing effective policymaking, both (inter)nationally and locally, to further protect children from the adverse effects of tobacco smoke.

2. METHODS

This protocol is reported according to the Preferred Reporting Items for Systematic Reviews and Meta-Analyses for Protocols (PRISMA-P) guideline for systematic review and meta-analysis protocols.[13]

Patient and public involvement

Patients and the public were not involved in the design, development, conduct, reporting, or dissemination of this study.

Eligibility criteria

We will include studies based on pre-defined criteria as summarised in Table 1 and detailed in the text below.

For peer review only - http://bmjopen.bmj.com/site/about/guidelines.xhtml

Table 1. Inclusion criteria based on PICOS strategy[14]

PICOS	Inclusion criteria	Exclusion criteria
**P**opulation	(1) 0- to 16-year-olds<br>(2) (Sub)populations in which at least 50% is under 17 years of age	(1) Adult population without a distinct child subgroup
**I**ntervention	Smoke-free policies instituted at any governmental or institutional level that restrict smoking in designated (semi)private places or/and any outdoor areas	Smoke-free policies covering enclosed public places
**C**omparison	(1) A comparison population living in a location where no intervention was introduced/changed in the observational period<br>(2) A comparison time period in which no intervention was introduced/changed	
**O**utcomes	I. Child TSE:<br>Primary outcome:<br>(1) TSE in places covered by the policy (as reported by child and/or parent/primary caregiver)<br>Secondary outcomes:<br>(1) TSE in places of which only some were covered by the policy or in unspecified places (as reported by child/parent/primary caregiver)<br>(2) TSE in places not covered by the policy (as reported by child/parent/primary caregiver)<br>(3) Cotinine or other specific biomarkers of TSE quantified in body fluids, hair, nails, or on the skin<br>(4) TSE assessed by wearable devices<br><br>II. Child health outcomes:<br>Primary outcomes:<br>(1) Unplanned hospital attendance for wheezing/asthma<br>(2) Unplanned hospital attendance for RTIs<br>Secondary outcomes:<br>(1) General incidence of wheezing/ asthma<br>(2) General incidence of RTI<br>(3) OME<br>(4) Chronic cough<br>(5) FEV1, FVC, FEV1/FVC ratio	(1) Child TSE outcomes that are not specific to tobacco smoke (e.g. $PM_{2.5}$ and CO)<br>(2) Outcomes that do not necessarily imply a change in child TSE (e.g. changes in tobacco smoke constituent level in a room)<br>(3) Outcomes assessing smoking initiation/cessation or smoking behaviour (e.g. among parents)
**S**tudy design	Included in the main analyses:<br>(1) Randomised trials<br>(2) Non-randomised trials<br>(3) Interrupted time series<br>(4) Controlled before-after studies<br><br>Included in sensitivity analyses:<br>(6) Prospective cohort studies<br>(7) Retrospective cohort studies<br>(8) Uncontrolled before-after studies	

Abbrevations: tobacco smoke exposure= TSE; respiratory tract infections= RTI; otitis media with effusion= OME; Lung function= FEV1, FVC, FEV1/FVC ratio; fine particulate matter= $PM_{2.5}$; carbon monoxide= CO

For peer review only - http://bmjopen.bmj.com/site/about/guidelines.xhtml

I. Population

Studies are eligible when they include children between 0 and 16 years. Any study reporting (sub)populations in which at least 50% fits this age criterion will also be eligible. Some studies may have included specific subgroups, for example, children with asthma.[15] We will include all studies that meet the inclusion criteria, irrespective of such additional restrictions in the populations.

II. Interventions

We will include studies that evaluated smoke-free policies that were introduced at any governmental level (e.g. cities, municipalities, regions, or countries) or any institutional level (e.g. school, hospital). The included policies might concern the following areas: indoor private places (e.g. cars), indoor semi-private places (e.g. multi-unit housing), outdoor (semi)private places (e.g. shared gardens), and outdoor public places (e.g. parks, school grounds, beaches, hospital grounds). We will exclude studies that evaluated policies covering enclosed public places (i.e. 'traditional' smoke-free polices).

Smoke-free policies can be a part of a complex reform package.[16] For example, the above described smoke-free policy might be introduced simultaneously with other tobacco control policies (e.g. increased taxes on tobacco products). We will include all studies that estimate the effect of smoke-free policies covering (semi)private or/and outdoor places irrespective of whether other policies were introduced at the same time. If the effect of the smoke-free policy covering (semi)private or/and outdoor places has been disentangled from other interventions, we will extract the effect estimates from the analyses that most closely reflects that of this smoke-free policy.

III. Comparison

We will include studies that estimate the counterfactual scenario (i.e. no change in smoke-free policy implemented) using any of the following comparators: (1) a comparison population where no change in the observed smoke-free policy occurred in the observational period, or (2) a comparison time period in which no change in the observed smoke-free policy occurred.

IV. Outcomes

We will include studies assessing the impact of the policy on two types of outcomes: (1) indicators of TSE and (2) respiratory health outcomes. Any study that reported step changes (immediate change in incidence) and/or slope changes (gradual change in incidence over time) in one or more of these outcomes will be eligible.

Studies that report on the following TSE indicators will be included:

Primary outcome:

(1) TSE in places covered by the policy (as reported by the child/parent/primary caregiver)

Secondary outcomes:

(1) TSE in places of which only some were covered by the policy, or in unspecified places (as reported by the child and/or parent/primary caregiver)
(2) TSE in places not covered by the policy (as reported by the child/parent/primary caregiver)
(3) Cotinine or other specific biomarkers of TSE quantified in body fluids, hair, nails, or on the skin
(4) TSE assessed by wearable devices

Note that the primary TSE outcome of interest assesses exposure specifically in places covered by the policy. That is, we aim to assess whether the policy was successful in reducing child TSE in those places that it was intended to cover. In addition, unintended effects of smoke-free policies on TSE have been described, both positive (spill-over; e.g. via increased adoption of voluntary home smoking bans following the implementation of smoke-free policy) and negative (displacement; e.g. via increased smoking at home following smoke-free

For peer review only - http://bmjopen.bmj.com/site/about/guidelines.xhtml

vehicle regulation)[11]. In order to identify such effects we will also identify studies that have assessed changes in TSE in places not covered by the policy.

Individual studies may report different estimates for TSE based on various definitions. We will prioritise child-reported TSE outcomes over parental or primary caregiver reported outcomes for studies reporting both. Furthermore, we will prioritise past-week TSE outcomes over longer or shorter recall periods. If past-week TSE is not available, longer recalls are prioritised over shorter recall periods.

The reduction in child TSE is anticipated to translate into child health benefits. Health outcomes are selected based on severity and responsiveness to changes in TSE within a reasonably short time frame (i.e. within one year), these primarily being respiratory outcomes.[2 12] Accordingly, we will include studies that report the following outcomes:

Primary outcomes:

(1) Unplanned hospital attendance for wheezing/asthma
(2) Unplanned hospital attendance for RTIs

Secondary outcomes:

(1) General incidence of wheezing/ asthma
(2) General incidence of RTI
(3) Otitis media with effusion (OME)
(4) Chronic cough
(5) Lung function (FEV1, FVC, FEV1/FVC ratio)

Unplanned hospital attendance may include acute presentations to the accident and emergency department as well as hospital admissions. For the secondary outcomes, respiratory diseases may be based on different case definitions. For studies reporting estimates according to various definitions we will prioritise according to the following hierarchy: (1) based on physician diagnosis, (2) based on medication use (not applicable for OME), and (3) self-reported.

We will not include perinatal outcomes in this review as many of the policies for which we anticipate to identify evidence are specifically aimed at protecting children, not pregnant women (e.g. smoke-free policies in playgrounds).

V. Study designs

We will follow the guidelines of the Cochrane Effective Practice and Organisation of Care (EPOC) group to select eligible study designs.[17] EPOC established criteria for high-quality methodologies in research. These include: (1) randomised trials, (2) non-randomised trials, (3) interrupted time series, and (4) controlled before-after studies. We will include studies that satisfy these criteria in our main analysis. Based on previous experiences, this restriction may sometimes lead to a low number of studies being eligible. In order to properly assess the available evidence in such instances, we will conduct sensitivity analyses by relaxing the inclusion criteria.[2 18] For the sensitivity analyses, we will additionally include (1) prospective cohort studies, (2) retrospective cohort studies, and (3) uncontrolled before-after studies.

VI. Further restrictions

There will be no restriction on publication date, the timeframe of the study, or the length of follow-up after the introduction of the policy. We will include every study that meets the above-mentioned criteria regardless of the language. Studies published in languages other than English will be translated. Google Translate will be used for this purpose, and we will consult with a translator if necessary.

For peer review only - http://bmjopen.bmj.com/site/about/guidelines.xhtml

VII. Report type

Our systematic review will include published studies in scientific journals as well as 'grey literature' (i.e. documents that are circulated in non-commercial academic channels or/and not indexed by major databases). Only full reports will be included in our review. Studies for which only an abstract was published will not be included since risk of bias for these studies cannot be adequately assessed. In these cases, however, we will contact the authors to ask if a full report is available.

Information sources

We will search for potential studies in the following electronic databases: (1) Embase.com, (2) Medline Ovid, (3) Web of Science, (4) PsycINFO Ovid, (5) CINAHL EBSCOhost, (6) Google Scholar, (7) IndMED, (8) KoreaMed, (9) EconLit, (10) WHO Global Health Library [including African Index Medicus [AIM], Latin America and the Caribbean Literature on Health Sciences (LILACS), Index Medicus for the Eastern Mediterranean Region [IMEMR], Index Medicus for South-East Asia Region [IMSEAR], Western Pacific Region Index Medicus [WPRIM]], (11) WHO Library Database [WHOLIS], (12) Scientific Electronic Library Online [SciELO], and (13) Paediatric Economic Database Evaluation (PEDE).

Search strategy

First, we will search for potentially relevant studies based on a search strategy that is the combination of MeSH terms and free text search. Our research team, including a librarian who is specialised in search strategy optimisation, has developed this search strategy. Search terms were tailored to each database. By means of an example, search terms used for Embase.com database are available in the Appendix. Search terms included four parts: (1) terms to identify smoke-free policies; (2) terms that identify children as the target population; (3) terms to identify asthma, wheezing, respiratory diseases, OME, chronic cough, lung function, or TSE as outcome; and (4) terms that exclude conference abstracts, letter to the editors, notes and editorials.

Second, we will search for additional studies by screening reference lists of included studies and their citations through Google Scholar. Moreover, we will contact with experts in the field to identify additional studies which may have been missed and any relevant ongoing or unpublished studies. Finally, we will update our search to add the most recent publications just before submitting the review for publication.

Study records

I. Data management

We will extract all records identified by the different sources into an EndNote Library, and use this software to automatically de-duplicate the collected records according to the method previously described.[19] Subsequently, we will manually identify and remove any duplicates that remain. At this stage, duplicates will be identified based on overlaps in the authors' names, the titles of the publication, the observed populations, the constructions of the treatment group and control groups, sample sizes, and the reported outcomes. In our final report, we will note the number of duplicates in the PRISMA flow-diagram.

II. Selection process

After the de-duplication process, we will select eligible papers from the remaining unique records. At the first step, two independent researchers will screen titles and the abstracts for eligibility. We will then obtain the full-text reports of studies that may fit eligibility criteria based on this assessment. At the next step, two independent researchers will assess eligibility based on the full-texts. Any discrepancies will be resolved after discussion with a third researcher. The involved researchers will not be blinded to information about the articles (e.g. authors' names and affiliations) at any stage.

III. Data collection process

Two researchers will independently extract data from the included studies to a customised data extraction form developed *a priori* that has been piloted using six eligible studies. After finishing the data extraction, they will confer

For peer review only - http://bmjopen.bmj.com/site/about/guidelines.xhtml

their results with each other and create one final file. Again, any discrepancies will be resolved after discussion with a third researcher. We will contact authors for any relevant missing data.

In case overlapping populations are analysed in multiple studies, we will include according to the following hierarchy the study that (1) has the lowest risk of bias (see risk-of-bias assessment below), (2) evaluates the most comprehensive policy, or (3) incorporates the largest sample size. In case one study reports multiple effect estimates for overlapping populations, we will select according to the following hierarchy the one derived from (1) the most adjusted model, (2) the longest observation period, (3) the most comprehensive policy, or (4) the largest treatment group.

Data items

From eligible studies we will extract the following items: (1) the first author's name, (2) his or her affiliation, (3) publication year, (4) type of publication (journal, book, dissertation etc.), (5) access information (URL or doi), (6) study design, (7) observational period (the beginning and end dates), (8) exact places covered by the policy, (9) whether the policy covers outdoor or indoor places, (10) whether the policy covers public or (semi) private, places, (11) timing of policy, (12) institution/government that initiated the policy, (13) country/location where the policy had been implemented, (14) compliance with the policy, (15) enforcement of the policy, (16) eligibility criteria for inclusion, (17) description of control group(s), (18) population at risk, (19) number of participants/events, (20) control and treatment group size, (21) characteristics of the population and the treatment groups (e.g. age, gender, and socioeconomic status), (22) data source(s) used for the study, (23) definition(s) of outcome measure(s), (24) controlled confounders (if applicable), (25) applied statistical techniques to draw inference, (26) number of clusters (if applicable), (27) cluster size (if applicable), (28) whether the results were adjusted for clustering (if clustered study), (29) whether the intra-class correlation coefficient is reported and what it is (if clustered study), (30) the number of drop-outs/missing values, (31) techniques for handling missing values, (32) pre-intervention population at risk(n) / events(n) / rates (%) of outcome variable(s), (33) post-intervention population at risk(n) / events(n) / rates (%) of outcome variable(s), (34) association between smoke-free policy and outcome(s) (coefficients, confidence intervals, and p-values), (35) any unintended effects (e.g. TSE displacement), (36) proportion of children exposed to TSE in the location covered by the smoke-free policy, before and after the implementation of the policy, (37) risk ratios of respiratory disease for different levels of TSE according to specific locations covered by the smoke-free policy, (38) bias assessment (see section *Risk of bias assessment*), (39) elements supporting causal inference (see section *Elements supporting causal inference*), (40) any conflict of interest reported by the authors, and (41) the funding source(s).

Outcomes and prioritisation

As previously detailed, we will identify studies evaluating the effects of smoke-free policies on children's (1) TSE and (2) health outcomes. See the description of outcomes and our prioritisation in the *Eligibly criteria* section.

Risk of bias assessment

For each individual result, we will assess risk of bias using standardised assessment tools. For randomised trials, we will use the *Quality Assessment Tool for Quantitative Studies,* developed by EPOC.[20] For non-randomised studies, we will use the *Risk Of Bias In Non-randomised Studies of Interventions* (ROBINS-I) tool.[21] The ROBINS-I tool evaluates biases in the following domains: (1) bias due to confounding (Table 2 in Appendix), (2) bias in selection of participants into the study, (3) bias in classification of interventions, (4) bias due to deviations from intended interventions (See Appendix), (5) bias due to missing data, (6) bias in measurement of outcomes, and (7) bias in selection of the reported result. For each domain, we will rate the risk of bias on a 4-point scale ranging from critical risk of bias to low risk of bias (i.e. bias is comparable to a well-performed randomised trial). The ROBINS-I tool will be followed to give an overall estimate of the risk of bias. The risk-of-bias assessments will be conducted independently by two researchers and a third reviewer will be consulted to resolve disagreements.

Elements supporting causal inference

We will make a further attempt to reflect on reported elements that may support causal inference and the robustness of the evidence using the UK Medical Research Council guidance on natural experiments.[22] First, we will report whether the effect estimates were robust when using alternative comparison groups (e.g. the use of different

For peer review only - http://bmjopen.bmj.com/site/about/guidelines.xhtml

populations living in distinct geographical areas as a comparison). Second, we will report whether a study estimated whether the smoke-free policy also had an 'effect' on neutral outcomes that were not expected to change as a consequence of the intervention (e.g. hospital admission for appendicitis). Finally, we will report whether complementing research methodologies (e.g. qualitative analyses) identified plausible underlying mechanisms supporting the main conclusions. These assessments will be based on the information presented in the study reports included in the review. The information presented in the published papers will be presented in tables, and a summary will be narratively described.

Data synthesis

I. Synthetisation of data

Obtaining comparable data is essential to facilitate meta-analysis. Studies should be sufficiently similar in terms of their designs and the definitions of their measurements. We will uniformise outcome assessments before conducting data analysis. First, if continuous outcomes appear on different scales across studies we will use standardised mean differences. If continuous outcomes appear on the same scale, weighted mean differences will be used. Second, for dichotomous outcomes we will extract relative risks. If only absolute risks are presented then we will calculate relative risks. Third, we will express the effects on dichotomous outcomes using risk ratio (RR). If RR was not reported, we will contact the authors to provide it to us. If the requested data was not be obtained, we will convert OR to RR using the following formula:

$$RR = \frac{OR}{(1 - PEER) + (PEER \times OR)}$$

, where $PEER$ refers to the patient-expected event rate in the control group.[23] In case $PEER$ is not available, we will approximate this measure using the overall event rate across the entire study population. We will use incidence rate ratios (IRRs) instead of RR for outcomes that could occur repeatedly with the same individual (i.e. hospitalisation). To make different TSE measures comparable, we will convert them into a binary variable with a value of 1 if the child was exposed to (detectable amounts of) TSE and 0 if unexposed to (detectable amounts of) TSE. Finally, two researchers will check independently whether the definitions of intervention, population, and outcomes are sufficiently comparable across the selected studies to allow meta-analysis.

The eligible studies are likely to be heterogeneous in various important characteristics which will be taken into account in the data synthesis. Therefore we will apply random-effects models (as opposed to fixed-effects) for data analysis when three or more studies evaluated similar policies and reported on the same outcome. We will separately pool step changes (immediate changes) and slope changes (gradual changes) for each outcome in different models. If unspecified, the result will be considered a step change. We will conduct separate meta-analyses for various subtypes of smoke-free policies, according to the locations that they cover. Thus we will separately pool studies for (1) smoke-free policies covering indoor private places (e.g. cars), (2) smoke-free policies covering indoor semi-private places (e.g. multi-unit housing), (3) smoke-free policies covering outdoor private or semi-private spaces (e.g. shared gardens), and (4) smoke-free policies covering outdoor public places (e.g. parks, school grounds, beaches, and hospital grounds). Finally, regarding TSE we will pool separate models to assess unintended effects of the policy in places not covered by the policy.

The estimations of our meta-analysis will be displayed in forest plots. Heterogeneity will be quantified by the I^2 statistic for each pooled model.

We anticipate that most studies will have assessed the impact of novel smoke-free policies on TSE rather than on health outcomes. As the relationship between TSE and respiratory outcomes among children is well established,[24 25] we will use data from studies estimating the impact of novel smoke-free policies on child TSE to quantify the potential impact of these changes on our primary health outcomes. Thus, we will perform a health impact assessment and quantify the proportion of children with respiratory disease that can be prevented by the change in TSE following smoke-free policies. First, we will extract the risk ratio of respiratory disease for those children who were exposed to

For peer review only - http://bmjopen.bmj.com/site/about/guidelines.xhtml

TSE versus those who were not either from the studies included in our systematic review or from previous meta-analyses that quantified this association.[24 25] Preferably, this risk ratio will be retrieved for the specific locations that are covered by the smoke-free policy and for distinct respiratory diseases. Second, based on the risk ratios and the difference in the proportion of children exposed to TSE before and after the policy implementation, we will calculate two Population Attributable Fractions (PAFs). One quantifies the proportion of each outcome that can be attributed to TSE before the smoke-free policy and the other after the policy's implementation. The difference of these PAFs will give us the Potential Impact Fraction (PIF) capturing the change in the outcomes when the TSE level changes following a novel smoke-free policy's implementation.

II. Sensitivity analyses

Two sets of sensitivity analyses will be conducted. First, excluding results with a higher risk of bias might lead to different estimations. Thus, we will conduct meta-regression analyses using the overall risk of bias based on the ROBINS-I assessment to test if effect sizes differ according to the overall risk of bias. Second, the question may arise whether a less restrictive selection strategy regarding study design would lead to different results. We will check for the robustness of our results by additionally including studies applying not only methodologies considered as the state-of-the-art in the *Cochrane EPOC guidelines*, but also study designs with a larger risk of bias.[17]

III. Subgroup analyses

We will conduct subgroup analyses to evaluate whether smoke-free policies have differential effects among certain subgroups. To assess whether policies introduced in high-income countries have a different effect than policies introduced in low- or middle-income countries, we will perform meta-regression based on the World Bank classification.[26] Second, previous studies have shown that smoke-free policies have a different effect according to individuals' SES.[18 27-29] We will extract information from subgroup analyses by SES indicators where possible and perform separate meta-analyses for high and low SES groups.

Meta-bias assessment

In each meta-analysis, we will assess the extent of publication bias via funnel plots when at least ten studies are available. Furthermore, we will look for unpublished studies for which a protocol was registered, and we will contact the authors of these studies to obtain their results. Subsequently, we will compare the findings of these unpublished studies with those of the published reports. Finally, we will look for published reports that followed a registered protocol. We will compare these protocols with the final papers to check whether selective reporting was present at any stage.

Confidence in cumulative estimate

We will discuss the strength of the evidence on the association between implementation of smoke-free policies covering (semi)private or/and outdoor places and the TSE and child respiratory health outcomes based on the effect sizes, the variances of the estimated effects, the estimated level of heterogeneity between studies, sensitivity analyses, subgroup analyses, risk of bias, elements supporting causal inference and publication bias.

3. ETHICS AND DISSEMINATION

Ethical approval will not be required because we will only be using aggregate-level published data from previous studies. We will publish our results in a peer-reviewed international journal and report the research findings according to the PRISMA guideline.[30] Finally, we will disseminate our results to the public and policy-makers following the acceptance of our paper (e.g. actively seek media-attention and interaction with policy-makers and other relevant stakeholders).

Contributors JVB secured funding for this work. All authors contributed to the conceptualisation of the review and developed the methods. MKR, FJM, and JVB drafted the manuscript and AS, CM, WB, AB, and FJL provided feedback on manuscript drafts. JVB and WB developed the search strategy. All authors approved the manuscript.

For peer review only - http://bmjopen.bmj.com/site/about/guidelines.xhtml

Funding This study is funded by a project grant from the Dutch Heart Foundation, Lung Foundation Netherlands, Dutch Cancer Society, Dutch Diabetes Research Foundation, and the Netherlands Thrombosis Foundation. The Public Health Policy Evaluation Unit at Imperial College London is grateful for the support of the NIHR School of Public Health Research.

Competing interests None declared.

Patient consent for publication Not required.

Acknowledgement: We thank Lauren Westenberg, Brigit Toebes and Martine Bouman for providing input.

REFERENCES

1. Öberg M, Jaakkola MS, Woodward A, et al. Worldwide burden of disease from exposure to second-hand smoke: a retrospective analysis of data from 192 countries. *Lancet* 2011;377(9760):139-46 doi:10.1016/S0140-6736(10)61388-8.
2. Faber T, Kumar A, Mackenbach JP, et al. Effect of tobacco control policies on perinatal and child health: a systematic review and meta-analysis. *c* 2017;2(9):420-37 doi:10.1016/S2468-2667(17)30144-5.
3. Toebes B, Gispen ME, Been JV, et al. A missing voice: the human rights of children to a tobacco-free environment. *Tob Control* 2018;27(1):3-5 doi:10.1136/tobaccocontrol-2017-053657.
4. Jarvis MJ, Sims M, Gilmore A, et al. Impact of smoke-free legislation on children's exposure to secondhand smoke: cotinine data from the Health Survey for England. *Tob Control* 2012;18(1):749-61 doi:10.1136/tc.2010.041608.
5. Akhtar PC, Currie DB, Currie CE, et al. Changes in child exposure to environmental tobacco smoke (CHETS) study after implementation of smoke-free legislation in Scotland: national cross sectional survey. *BMJ* 2007;335(7619):545.49 doi:10.1136/bmj.39311.550197.AE.
6. Holliday JC, Moore GF, Moore LA. Changes in child exposure to secondhand smoke after implementation of smoke-free legislation in Wales: a repeated cross-sectional study. *BMC Public Health* 2009;9(1):430 doi:10.1186/1471-2458-9-430.
7. Sims M, Bauld L, Gilmore A. England's legislation on smoking in indoor public places and work-places: impact on the most exposed children. *Addiction* 2012;107(11):2009-16 doi:10.1111/j.1360-0443.2012.03924.x.
8. Nanninga S, Lhachimi SK, Bolte G. Impact of public smoking bans on children's exposure to tobacco smoke at home: a systematic review and meta-analysis. *BMC Public Health* 2018;18(1):749 doi:10.1186/s12889-018-5679-z.
9. Painter K. Outdoor smoking: fair or foul? *BMJ* 2019;366:l5754 doi:10.1136/bmj.l5754.
10. Laverty AA, Hone T, Vamos EP, et al. Impact of banning smoking in cars with children on exposure to second-hand smoke: a natural experiment in England and Scotland. *Thorax* 2020 doi:10.1136/thoraxjnl-2019-213998.
11. Faber T, Mizani MA, Sheikh A, et al. Investigating the effect of England's smoke-free private vehicle regulation on changes in tobacco smoke exposure and respiratory disease in children: a quasi-experimental study. *Lancet Public Health* 2019;4(12):607-17 doi:10.1016/S2468-2667(19)30175-6.
12. Been JV, Nurmatov UB, Cox B, et al. Effect of smoke-free legislation on perinatal and child health: a systematic review and meta-analysis. *Lancet* 2014;383(9928):1549-60 doi:10.1016/S0140-6736(14)60082-9.
13. Shamseer L, Moher D, Clarke M, et al. Preferred reporting items for systematic review and meta-analysis protocols (PRISMA-P) 2015: elaboration and explanation. *BMJ* 2015; 4(1):1 doi:10.1136/bmj.g7647.
14. O'Connor D, Green S, Higgins JPT. Defining the review question and developing criteria for including studies. Cochrane handbook for systematic reviews of interventions 2008 doi:10.1002/9780470712184.ch5.
15. Croghan IT, Ebbert JO, Hays JT, et al. Impact of a countywide smoke-free workplace law on emergency department visits for respiratory diseases: a retrospective cohort study. *BMC Pulm Med* 2015;15(1):6 doi:10.1186/1471-2466-15-6.
16. World Health Organization. WHO report on the global tobacco epidemic, 2017: monitoring tobacco use and prevention policies. https://www.who.int/tobacco/global_report/2017/en/ (Accessed: 02.02.2020): World Health Organization 2017.
17. Higgins JP, Green S. Cochrane Handbook for Systematic Reviews of Interventions Version 6. 2018.
18. Been JV, Millett C, Lee JT, et al. Smoke-free legislation and childhood hospitalisations for respiratory tract infections. *Eur Respir J* 2015;46(3):697-706 doi:10.1183/09031936.00014615.

For peer review only - http://bmjopen.bmj.com/site/about/guidelines.xhtml

19. Bramer WM, Giustini D, de Jonge GB, et al. De-duplication of database search results for systematic reviews in EndNote. *J Med Libr Assoc* 2016;104(3):240-43 doi:10.3163/1536-5050.104.3.014.
20. Effective Public Health PracticeProject (EPHPP). Quality Assessment Tool for Quantitative Studies. 2010.
21. Sterne JA, Hernán MA, Reeves BC, et al. ROBINS-I: a tool for assessing risk of bias in non-randomised studies of interventions. *BMJ* 2016;355:i4919 doi:10.1136/bmj.i4919.
22. Craig P, Cooper C, Gunnell D, et al. Using natural experiments to evaluate population health interventions: new Medical Research Council guidance. *J Epidemiol Community Health* 2012;66(12):1182-86 doi:10.1136/jech-2011-200375.
23. Cummings P. The relative merits of risk ratios and odds ratios. *Arch Pediatr Adolesc Med* 2009;163(5):438-45doi:10.1001/archpediatrics.2009.31.
24. Vardavas CI, Hohmann C, Patelarou E. The independent role of prenatal and postnatal exposure to active and passive smoking on the development of early wheeze in children. *Eur Respir J* 2016;48(1):115–24 doi:10.1183/13993003.01016-2015.
25. Jones LL, Hashim A, McKeever T, et al. Parental and household smoking and the increased risk of bronchitis, bronchiolitis and other lower respiratory infections in infancy: systematic review and meta-analysis. *Respir Res* 2011;12(1):5 doi:10.1186/1465-9921-12-5.
26. World Bank. World Bank Open Data: Access: https://data.worldbank.org/ (Downloaded: 07.09.2019), 2019 doi:.
27. Hajdu T, Hajdu G. Smoking ban and health at birth: Evidence from Hungary. *Econ Hum Biol* 2018;30:37-47 doi:10.1016/j.ehb.2018.05.003.
28. Vicedo-Cabrera AM, Schindler C, Radovanovic D, et al. Benefits of smoking bans on preterm and early-term births: a natural experimental design in Switzerland. *Tob Control* 2016;25(2):135-41 doi:10.1136/tobaccocontrol-2015-052739.
29. Bakolis I, Kelly R, Fecht D, et al. Protective effects of smoke-free legislation on birth outcomes in England-a regression discontinuity design. *Epidemiology* 2016;27(6):810-18 doi:10.1097/EDE.0000000000000534.
30. Liberati A, Altman DG, Tetzlaff J, et al. The PRISMA statement for reporting systematic reviews and meta-analyses of studies that evaluate health care interventions: explanation and elaboration. *PLoS Med* 2009;6(7) doi:10.1371/journal.pmed.1000100.

For peer review only

For peer review only - http://bmjopen.bmj.com/site/about/guidelines.xhtml

Appendix:

Search terms used for Embase.com database

('smoking regulation'/exp OR 'smoking ban'/de OR (smokefree OR ((smoking OR smoke OR tobacco) NEAR/3 (regulation* OR government* OR law OR laws OR policy OR policies OR ban OR bans OR banned OR free OR restrict* OR act OR acts))):ab,ti) AND (child/exp OR pediatrics/exp OR childhood/exp OR 'child welfare'/de OR 'child development'/de OR 'child health'/de OR 'child health care'/exp OR 'child care'/exp OR 'childhood disease'/exp OR 'pediatric ward'/de OR 'pediatric hospital'/de OR (infan* OR child* OR kid OR kids OR toddler* OR boy* OR girl* OR minors OR underag* OR (under NEXT/1 (age* OR aging)) OR kindergar* OR prepubescen* OR prepubert* OR pediatric* OR paediatric* OR school* OR preschool* OR (age NEAR/3 analy*)):ab,ti) AND ('asthma'/exp OR 'wheezing'/exp OR 'respiratory tract infection'/exp OR 'coughing'/exp OR 'otitis media'/exp OR 'exposure'/de OR 'self report'/exp OR cotinine/exp OR nicotine/exp OR 'saliva analysis'/exp OR 'urinalysis'/exp OR 'electronic device'/de OR 'wearable device'/de OR 'wearable sensor'/de OR 'hair analysis'/exp OR 'skin analysis device'/de OR 'passive smoking'/de OR 'environmental exposure'/de OR 'lung function test'/exp OR 'lung volume'/exp OR (asthma* OR wheezing OR ((respirator* OR lung OR pulmonar* OR expirat*) NEAR/3 (infection* OR function* OR volume*)) OR pneumonia OR bronchiolitis* OR cough* OR (otitis NEAR/3 media) OR exposure* OR environment* OR self-report* OR parent*-report* OR carer*-report* OR nicotine OR cotinin* OR second-hand* OR third-hand* OR secondhand* OR thirdhand* OR (passive NEAR/3 smoking) OR ((urine* OR saliva* OR hair OR hand* OR skin) NEAR/3 analy*) OR urinalys* OR ((electronic OR wearable) NEAR/3 (device* OR sensor*)) OR spirometr* OR fev OR fev1 OR fvc OR (forced NEAR/3 vital NEAR/3 capacit*) OR Tiffeneau):ab,ti) NOT ([Conference Abstract]/lim OR [editorial]/lim OR [letter]/lim)

Bias assessment criteria (confounders and co-interventions)

ROBINS-I requires to pre-specify certain risk-of-bias criteria that are relevant for the given study. For assessing the risk of bias due to confounding, we consider the following potential confounders relevant: age, gender, socio-economic status (SES) and/or ethnicity, and the pre-implementation temporal trend in the given outcome variable. We will score results as having a low risk of bias due to confounding if it controls for all of these variables, moderate risk of bias if not all confounders are controlled for but the pre-implementation trend is, high risk of bias if the pre- implementation trend is not controlled for but some of the other confounders are, and critical risk of bias if none of the listed confounders are controlled for. Other potential contextual confounders specifically for the child health outcomes are air pollution, quality of the health care system, access to health care facilities, and the vaccination programme in the country. Therefore, additionally to the criteria noted above, studies will also be considered to have a critical risk of bias due to confounding if contextual confounders were unaccounted for despite these being reported to be an issue in the study.

For assessing bias due to co-interventions (i.e. part of 'bias due to deviations from intended interventions'), the following co-interventions are considered: policies aimed at reducing ambient air pollution, introduction of relevant health-care reforms and/or public health programmes, introduction of other tobacco control policies, change in vaccination programmes, and wars/epidemics/economic crises which influenced the health of children. We will approximate the percentage of the observation units affected by any of these uncontrolled co-interventions. When this percentage is below 10% a result will be considered to have a low risk of bias, when it ranges between 10% and 20% moderate risk of bias, between 20% and 40% high risk of bias, and above 40% it will be considered serious risk of bias.

For peer review only - http://bmjopen.bmj.com/site/about/guidelines.xhtml

Table 2. Bias due to confounding and due to deviations from intended interventions

Level of bias	Bias due to confounding	Bias due to deviations from intended interventions
**Low risk of bias**	(I) The pre-implementation temporal trends in the outcomes are controlled<br>(II) Individual characteristics are controlled<br>(III) (For health outcomes): Contextual characteristics are controlled if reported to be an issue in the study	The percentage of the units affected by any of the co-interventions is below 10%
**Moderate risk of bias**	(I) The pre-implementation temporal trends in the outcomes are controlled<br>(II) Individual characteristics are not all controlled<br>(III) (For health outcome): Contextual characteristics are controlled if reported to be an issue in the study	The percentage of the units affected by any of co-interventions is between 10% and 20%
**Serious risk of bias**	(I) The pre-implementation temporal trends in the outcomes are not controlled<br>(II) Some of the individual characteristics are controlled<br>(III) (For health outcome): Contextual characteristics are controlled if reported to be an issue in the study	The percentage of the units affected by any of co-interventions is between 20% and 40%
**Critical risk of bias**	(I) The pre-implementation temporal trends in the outcomes are not controlled<br>(II) Some of individual characteristics are controlled<br>**OR**<br>(III) (FOR health outcome): Contextual characteristics are not controlled while being reported to be an issue in the study	The percentage of the units affected by any of co-interventions is above 40%
**No information**	No information on the controlled confounding variables	No information on the controlled co-interventions

For peer review only - http://bmjopen.bmj.com/site/about/guidelines.xhtml

BMJ Open

Impact of expanding smoke-free policies beyond enclosed public places and workplaces on children's tobacco smoke exposure and respiratory health: protocol for a systematic review and meta-analysis

Journal:	*BMJ Open*
Manuscript ID	bmjopen-2020-038234.R1
Article Type:	Protocol
Date Submitted by the Author:	21-Jul-2020

Complete List of Authors:	Rado, Márta; Erasmus MC Sophia, Department of Paediatrics; Erasmus MC, Department of Public Health<br>Mölenberg, Famke; Erasmus MC, Department of Public Health<br>Sheikh, Aziz; The University of Edinburgh Division of Community Health Sciences; Harvard Medical School, Brigham and Women's Hospital<br>Millett, Christopher; Imperial College London Department of Primary Care and Social Medicine<br>Bramer, Wichor; Erasmus MC, Medical Library<br>Burdorf, Alex; Erasmus MC, Department of Public Health<br>Van Lenthe, Frank; Erasmus MC, Department of Public Health<br>Been, JV; Erasmus MC Sophia, Department of Paediatrics; Erasmus MC, Department of Public Health

**Primary Subject Heading**:	Health policy
Secondary Subject Heading:	Epidemiology, Paediatrics, Public health, Smoking and tobacco
Keywords:	Health policy < HEALTH SERVICES ADMINISTRATION & MANAGEMENT, Child protection < PAEDIATRICS, Community child health < PAEDIATRICS, PUBLIC HEALTH, Respiratory infections < THORACIC MEDICINE

SCHOLARONE™
Manuscripts

I, the Submitting Author has the right to grant and does grant on behalf of all authors of the Work (as defined in the below author licence), an exclusive licence and/or a non-exclusive licence for contributions from authors who are: i) UK Crown employees; ii) where BMJ has agreed a CC-BY licence shall apply, and/or iii) in accordance with the terms applicable for US Federal Government officers or employees acting as part of their official duties; on a worldwide, perpetual, irrevocable, royalty-free basis to BMJ Publishing Group Ltd ("BMJ") its licensees and where the relevant Journal is co-owned by BMJ to the co-owners of the Journal, to publish the Work in this journal and any other BMJ products and to exploit all rights, as set out in our licence.

The Submitting Author accepts and understands that any supply made under these terms is made by BMJ to the Submitting Author unless you are acting as an employee on behalf of your employer or a postgraduate student of an affiliated institution which is paying any applicable article publishing charge ("APC") for Open Access articles. Where the Submitting Author wishes to make the Work available on an Open Access basis (and intends to pay the relevant APC), the terms of reuse of such Open Access shall be governed by a Creative Commons licence – details of these licences and which Creative Commons licence will apply to this Work are set out in our licence referred to above.

Other than as permitted in any relevant BMJ Author's Self Archiving Policies, I confirm this Work has not been accepted for publication elsewhere, is not being considered for publication elsewhere and does not duplicate material already published. I confirm all authors consent to publication of this Work and authorise the granting of this licence.

For peer review only

Impact of expanding smoke-free policies beyond enclosed public places and workplaces on children's tobacco smoke exposure and respiratory health: protocol for a systematic review and meta-analysis

Authors: Marta K. Rado [ab], Famke J.M. Mölenberg [b], Aziz Sheikh [cd], Christopher Millett [e], Wichor M. Bramer [f], Alex Burdorf [b], Frank J. van Lenthe [b], Jasper V. Been [abc] *

[a] Division of Neonatology, Department of Paediatrics, Erasmus MC — Sophia Children's Hospital, University Medical Centre Rotterdam, Rotterdam, Netherlands

[b] Department of Public Health, Erasmus MC, University Medical Centre Rotterdam, Rotterdam, Netherlands

[c] Centre of Medical Informatics, Usher Institute, The University of Edinburgh, Edinburgh, UK

[d] Division of General Internal Medicine and Primary Care, Brigham and Women's Hospital/Harvard Medical School, Boston, MA, USA

[e] Public Health Policy Evaluation Unit, School of Public Health, Imperial College London, London, UK

[f] Medical Library, Erasmus MC, University Medical Centre Rotterdam, Rotterdam, Netherlands

*To whom correspondence should be addressed:

Dr Jasper V. Been, MD MPH PhD

Erasmus MC— Sophia Children's Hospital,

Rotterdam, Netherlands PO Box 2060, 3000 CB Rotterdam

Phone: +31-10-7037048

Email: j.been@erasmusmc.nl

Keywords: smoke free policy, child health, tobacco smoke, respiratory health, meta-analysis

Word count: 4810

For peer review only

ABSTRACT

Introduction: Tobacco smoke exposure (TSE) has considerable adverse respiratory health impact among children. Smoke-free policies covering enclosed public places are known to reduce child TSE and benefit child health. An increasing number of jurisdictions are now expanding smoke-free policies to also cover outdoor areas and/or (semi-) private spaces (indoor and/or outdoor). We aim to systematically review the evidence on the impact of these 'novel smoke-free policies' on children's TSE and respiratory health.

Methods and analysis: 13 electronic databases will be searched by two independent reviewers for eligible studies. We will consult experts from the field and hand-search references and citations to identify additional published and unpublished studies. Study designs recommended by the Cochrane Effective Practice and Organisation of Care (EPOC) group are eligible, without restrictions on the observational period, publication date, or language. Our primary outcomes are: self- or parental-reported TSE in places covered by the policy; unplanned hospital attendance for wheezing/asthma; and unplanned hospital attendance for respiratory infections. We will assess risk of bias of individual studies following the EPOC or ROBINS-I tool, as appropriate. We will conduct separate random-effects meta-analyses for smoke-free policies covering (1) indoor private places, (2) indoor semi-private places, (3) outdoor (semi)private places, and (4) outdoor public places. We will assess whether the policies were associated with changes in TSE in other locations (e.g. displacement). Subgroup analyses will be conducted based on country income classification (i.e. high, low or middle-income) and by socioeconomic status (SES). Sensitivity analyses will be undertaken via broadening our study design eligibility criteria (i.e. including non-EPOC designs) or via excluding studies with a high risk of bias. This review will inform policymakers regarding the implementation of extended smoke-free policies to safeguard children's health.

Ethics and dissemination: Ethical approval is not required. Findings will be disseminated to the academics and the general public.

PROSPERO registration number: CRD42020190563

Strengths and limitations of this study

- This systematic review will be the first to comprehensively synthesise the evidence regarding the impact of 'novel smoke-free policies' (i.e. those covering (1) indoor private places, (2) indoor semi-private places, (3) outdoor (semi)private places, and (4) outdoor public places).
- This protocol has been designed in line with the Preferred Reporting Items for Systematic Reviews and Meta-Analyses for Protocols (PRISMA-P) guidelines and guidelines of the Cochrane Effective Practice and Organisation of Care (EPOC)
- A possible limitation is that included studies might be heterogeneous in the policy, study design and data collection methods which might limit our ability to synthesise the results using meta-analysis
- The value of this systematic review depends on the quality and availability of the evidence on the topic
- This work will be instrumental in informing effective policymaking regarding implementing novel smoke-free policies to protect children from the adverse effects of tobacco smoke.

For peer review only - http://bmjopen.bmj.com/site/about/guidelines.xhtml

1. INTRODUCTION

Tobacco smoke exposure (TSE) is an important cause of adverse respiratory health outcomes in children worldwide. Tobacco smoke exposure includes both second-hand smoke (SHS) and third-hand smoke (THS) exposure. SHS refers to the inhalation of emitted smoke, while THS exposure refers to the uptake of tobacco smoke residuals from polluted surfaces after someone has finished smoking. TSE is estimated to cause 166,000 child deaths annually associated with an increased risk of respiratory tract infections (RTI), wheezing, and asthma.[1 2]

Safeguarding of child health provides justification to advocate for strong tobacco control measures.[3] Previous systematic reviews found that smoke-free policies in enclosed public places are successful in reducing adverse child health outcomes. Meta-analyses showed that these policies were followed by a 9.8% (95% confidence interval [CI] 3.0 – 16.6) reduction in hospital attendance for asthma exacerbations, and an 18.5% (95% CI 4.2– 32.8) reduction in hospital attendance for lower RTIs.[2] These health impacts of smoke-free policies are likely mediated through a reduction in SHS and potentially also in THS.[1] Indeed, individual studies have demonstrated that smoke-free policies covering enclosed public areas are successful in decreasing child TSE in public places and even in private places, such as cars or homes, most likely via changes in social norms.[4-8] Although in low and middle-income countries high background air pollution, poor economic conditions, and low awareness of tobacco-related harm might obscure the positive effects of smoke-free legislation, evidence suggests that smoke-free policies can have a similarly positive impact in these countries as in high-income countries.[9 10]

As a result, smoke-free legislation is increasingly recognised as an important policy tool to protect children from the adverse health effects of TSE (e.g. incorporated in Sustainable Development Goal 3.2, 3.4, 3.9, and 3.A that aims to improve health and well-being).[3] Recently, policies to provide additional protection of children to TSE beyond enclosed public places have been implemented in a number of places, both at a national and a sub-national level. These include expansion to cover outdoor areas frequented by children such as school grounds, playgrounds and parks.[11] Also, some countries and subnational regions have now implemented policies prohibiting smoking in semi-private spaces such as shared housing or private spaces such as cars.[12 13]

The positive effects of smoke-free policies covering enclosed public places on child health are now well established.[2 14] A systematic synthesis of the evidence on the effectiveness of smoke-free policies covering outdoor and/or (semi)private spaces to reduce children's TSE and benefit their health, is however currently lacking.[2 14] It is important to note that effectiveness of smoke-free policies covering indoor public places cannot be easily extrapolated to those covering outdoor or private spaces, due to various reasons (e.g. dilution of TSE in outdoor spaces, enforcement issues). Further, evidence from various countries indicates that smoke-free policies covering public enclosed places and workplaces are followed by reductions in TSE even in areas not covered by the policy through norm spreading. Whether novel smoke-free policies also have an impact on TSE in places not covered by the policy is unclear. Theoretically, there may be a reduction (via norm spreading) in such places but there may also be displacement of smoking to areas not covered by the policy.[13] Finally, effectiveness of smoke-free policies is likely to vary according to local rates of smoking and children's SHS exposure, and comprehensiveness of the policy as well as compliance and enforcement.[15]

With an increasing number of institutions and governments now implementing smoke-free policies covering areas other than enclosed public spaces, optimal understanding of the effectiveness of such policies in protecting children is essential. To establish this evidence base, we will undertake a comprehensive systematic review of available studies assessing the impact of policies covering outdoor areas or/and (semi)private places (whether indoor or outdoor) on children's TSE and respiratory health. We believe this work will be instrumental to informing effective policymaking, both (inter)nationally and locally, to further protect children from the adverse effects of tobacco smoke.

2. METHODS

This protocol is reported according to the Preferred Reporting Items for Systematic Reviews and Meta-Analyses for Protocols (PRISMA-P) guideline for systematic review and meta-analysis protocols.[16]

Patient and public involvement

For peer review only - http://bmjopen.bmj.com/site/about/guidelines.xhtml

Patients and the public were not involved in the design, development, conduct, reporting, or dissemination of this study.

Eligibility criteria

We will include studies based on pre-defined criteria as summarised in Table 1 and detailed in the text below.[17]

For peer review only - http://bmjopen.bmj.com/site/about/guidelines.xhtml

Table 1. Inclusion criteria based on PICOS strategy

PICOS	Inclusion criteria	Exclusion criteria
**P**opulation	(1) 0- to 16-year-olds<br>(2) (Sub)populations in which at least 50% is under 17 years of age	(1) Adult population without a distinct child subgroup
**I**ntervention	Smoke-free policies instituted at any governmental or institutional level that restrict smoking in designated (semi)private places or/and any outdoor areas	Smoke-free policies covering enclosed public places
**C**omparison	(1) A comparison population living in a location where no intervention was introduced/changed in the observational period<br>(2) A comparison time period in which no intervention was introduced/changed	
**O**utcomes	I. Child TSE:<br>Primary outcome:<br>(1) TSE in places covered by the policy (as reported by child and/or parent/primary caregiver)<br>Secondary outcomes:<br>(1) TSE in places of which only some were covered by the policy or in unspecified places (as reported by child/parent/primary caregiver)<br>(2) TSE in places not covered by the policy (as reported by child/parent/primary caregiver)<br>(3) Cotinine or other specific biomarkers of TSE quantified in body fluids, hair, nails, or on the skin<br>(4) TSE assessed by wearable devices<br><br>II. Child health outcomes:<br>Primary outcomes:<br>(1) Unplanned hospital attendance for wheezing/asthma<br>(2) Unplanned hospital attendance for RTIs<br>Secondary outcomes:<br>(1) General incidence of wheezing/ asthma<br>(2) General incidence of RTI<br>(3) OME<br>(4) Chronic cough<br>(5) FEV1, FVC, FEV1/FVC ratio	(1) Child TSE outcomes that are not specific to tobacco smoke (e.g. $PM_{2.5}$ and CO)<br>(2) Outcomes that do not necessarily imply a change in child TSE (e.g. changes in tobacco smoke constituent level in a room)<br>(3) Outcomes assessing smoking initiation/cessation or smoking behaviour (e.g. among parents)
**S**tudy design	Included in the main analyses:<br>(1) Randomised trials<br>(2) Non-randomised trials<br>(3) Interrupted time series<br>(4) Controlled before-after studies<br><br>Included in sensitivity analyses:<br>(6) Prospective cohort studies<br>(7) Retrospective cohort studies<br>(8) Uncontrolled before-after studies	

Abbrevations: tobacco smoke exposure= TSE; respiratory tract infections= RTI; otitis media with effusion= OME; Lung function= FEV1, FVC, FEV1/FVC ratio; fine particulate matter= $PM_{2.5}$; carbon monoxide= CO

For peer review only - http://bmjopen.bmj.com/site/about/guidelines.xhtml

I. Population

Studies are eligible when they include children between 0 and 16 years. Any study reporting (sub)populations in which at least 50% fits this age criterion will also be eligible. Some studies may have included specific subgroups, for example, children with asthma.[18] We will include all studies that meet the inclusion criteria, irrespective of such additional restrictions in the populations.

II. Interventions

We will include studies that evaluated smoke-free policies that were introduced at any governmental level (e.g. cities, municipalities, regions, or countries) or any institutional level (e.g. school, hospital). The included policies might concern the following areas: indoor private places (e.g. cars), indoor semi-private places (e.g. multi-unit housing), outdoor (semi)private places (e.g. shared gardens), and outdoor public places (e.g. parks, school grounds, beaches, hospital grounds). We will exclude studies that solely evaluated policies covering enclosed public places (i.e. 'traditional' smoke-free polices).

Smoke-free policies can be a part of a complex reform package.[19] For example, the above described smoke-free policy might be introduced simultaneously with other tobacco control policies (e.g. increased taxes on tobacco products). We will include all studies that estimate the effect of smoke-free policies covering (semi)private or/and outdoor places irrespective of whether other policies were introduced at the same time. If the effect of the smoke-free policy covering (semi)private or/and outdoor places has been disentangled from other interventions, we will extract the effect estimates from the analyses that most closely reflects that of this smoke-free policy.

III. Comparison

We will include studies that estimate the counterfactual scenario (i.e. no change in smoke-free policy implemented) using any of the following comparators: (1) a comparison population where no change in the observed smoke-free policy occurred in the observational period, or (2) a comparison time period in which no change in the observed smoke-free policy occurred.

IV. Outcomes

We will include studies assessing the impact of the policy on two types of outcomes: (1) indicators of TSE and (2) respiratory health outcomes. We have specified a wide range of outcomes to obtain a comprehensive overview of the entirety of available evidence on the topic. We will in our interpretation focus primarily on a small set of primary outcomes next to a larger set of secondary outcomes. Any study that reported step changes (immediate change in incidence) and/or slope changes (gradual change in incidence over time) in one or more of these outcomes will be eligible.

Studies that report on the following TSE indicators will be included:

Primary outcome:

(1) TSE in places covered by the policy (as reported by the child/parent/primary caregiver)

Secondary outcomes:

(1) TSE in places of which only some were covered by the policy, or in unspecified places (as reported by the child and/or parent/primary caregiver)
(2) TSE in places not covered by the policy (as reported by the child/parent/primary caregiver)
(3) Cotinine or other specific biomarkers of TSE quantified in body fluids, hair, nails, or on the skin
(4) TSE assessed by wearable devices

Note that the primary TSE outcome of interest assesses exposure specifically in places covered by the policy. That is, we aim to assess whether the policy was successful in reducing child TSE in those places that it was intended to cover. In addition, unintended effects of smoke-free policies on TSE have been described, both

For peer review only - http://bmjopen.bmj.com/site/about/guidelines.xhtml

positive (spill-over; e.g. via increased adoption of voluntary home smoking bans following the implementation of smoke-free policy) and negative (displacement; e.g. via increased smoking at home following smoke-free vehicle regulation)[13]. In order to identify such effects we will also identify studies that have assessed changes in TSE in places not covered by the policy.

Individual studies may report different estimates for TSE based on various definitions. We will prioritise child-reported TSE outcomes over parental or primary caregiver reported outcomes for studies reporting both. Furthermore, we will prioritise past-week TSE outcomes over longer or shorter recall periods. If past-week TSE is not available, longer recalls are prioritised over shorter recall periods.

The reduction in child TSE is anticipated to translate into child health benefits. Health outcomes are selected based on severity and responsiveness to changes in TSE within a reasonably short time frame (i.e. within one year), these primarily being respiratory outcomes.[2 14] Accordingly, we will include studies that report the following outcomes:

Primary outcomes:

(1) Unplanned hospital attendance for wheezing/asthma
(2) Unplanned hospital attendance for RTIs

Secondary outcomes:

(1) General incidence of wheezing/ asthma
(2) General incidence of RTI
(3) Otitis media with effusion (OME)
(4) Chronic cough
(5) Lung function (FEV1, FVC, FEV1/FVC ratio)

Unplanned hospital attendance may include acute presentations to the accident and emergency department as well as hospital admissions. For the secondary outcomes, respiratory diseases may be based on different case definitions. For studies reporting estimates according to various definitions we will prioritise according to the following hierarchy: (1) based on physician diagnosis, (2) based on medication use (not applicable for OME), and (3) self-reported.

We will not include perinatal outcomes in this review as many of the policies for which we anticipate to identify evidence are specifically aimed at protecting children, not pregnant women (e.g. smoke-free policies in playgrounds).

V. Study designs

We will follow the guidelines of the Cochrane Effective Practice and Organisation of Care (EPOC) group to select eligible study designs.[20] EPOC established criteria for high-quality methodologies in research. These include: (1) randomised trials, (2) non-randomised trials, (3) interrupted time series, and (4) controlled before-after studies. We will include studies that satisfy these criteria in our main analysis. Based on previous experiences, this restriction may sometimes lead to a low number of studies being eligible. In order to properly assess the available evidence in such instances, we will conduct sensitivity analyses by relaxing the inclusion criteria.[2 21] For the sensitivity analyses, we will additionally include (1) prospective cohort studies, (2) retrospective cohort studies, and (3) uncontrolled before-after studies.

VI. Further restrictions

There will be no restriction on publication date, the timeframe of the study, or the length of follow-up after the introduction of the policy. All databases will be searched from their date of inception, and we will update the search to supplement our review just before publication. We will include every study that meets the above-mentioned criteria regardless of the language. Studies published in languages other than English will be translated. Google Translate will be used for this purpose, and we will consult with a translator if necessary.

For peer review only - http://bmjopen.bmj.com/site/about/guidelines.xhtml

VII. Report type

Our systematic review will include published studies in scientific journals as well as 'grey literature' (i.e. documents that are circulated in non-commercial academic channels or/and not indexed by major databases). Only full reports will be included in our review. Studies for which only an abstract was published will not be included since risk of bias for these studies cannot be adequately assessed. In these cases, however, we will contact the authors to ask if a full report is available.

Information sources

We will search for potential studies in the following electronic databases: (1) Embase.com, (2) Medline Ovid, (3) Web of Science, (4) PsycINFO Ovid, (5) CINAHL EBSCOhost, (6) Google Scholar, (7) IndMED, (8) KoreaMed, (9) EconLit, (10) WHO Global Health Library (including African Index Medicus [AIM], Latin America and the Caribbean Literature on Health Sciences [LILACS], Index Medicus for the Eastern Mediterranean Region [IMEMR], Index Medicus for South-East Asia Region [IMSEAR], Western Pacific Region Index Medicus [WPRIM]), (11) WHO Library Database [WHOLIS], (12) Scientific Electronic Library Online (SciELO), and (13) Paediatric Economic Database Evaluation (PEDE).

Search strategy

First, we will search for potentially relevant studies based on a search strategy that is the combination of MeSH terms and free text search. Our research team, including a librarian who is specialised in search strategy optimisation, has developed this search strategy. Search terms were tailored to each database. By means of an example, search terms used for Embase.com database are available in the Appendix. Search terms included four parts: (1) terms to identify smoke-free policies; (2) terms that identify children as the target population; (3) terms to identify asthma, wheezing, respiratory diseases, OME, chronic cough, lung function, or TSE as outcome; and (4) terms that exclude conference abstracts, letter to the editors, notes and editorials.

Second, we will search for additional studies by screening reference lists of included studies and their citations through Google Scholar. Moreover, we will contact with experts in the field to identify additional studies which may have been missed and any relevant ongoing or unpublished studies. Finally, we will update our search to add the most recent publications just before submitting the review for publication.

Study records

I. Data management

We will extract all records identified by the different sources into an EndNote Library, and use this software to automatically de-duplicate the collected records according to the method previously described.[22] Subsequently, we will manually identify and remove any duplicates that remain. At this stage, duplicates will be identified based on overlaps in the authors' names, the titles of the publication, the observed populations, the constructions of the treatment group and control groups, sample sizes, and the reported outcomes. In our final report, we will note the number of duplicates in the PRISMA flow-diagram.

II. Selection process

After the de-duplication process, we will select eligible papers from the remaining unique records. At the first step, two independent researchers will screen titles and the abstracts for eligibility. We will then obtain the full-text reports of studies that may fit eligibility criteria based on this assessment. At the next step, two independent researchers will assess eligibility based on the full-texts. Any discrepancies will be resolved after discussion with a third researcher. The involved researchers will not be blinded to information about the articles (e.g. authors' names and affiliations) at any stage.

III. Data collection process

Two researchers will independently extract data from the included studies to a customised data extraction form developed *a priori* that has been piloted using six eligible studies. After finishing the data extraction, they will confer

For peer review only - http://bmjopen.bmj.com/site/about/guidelines.xhtml

their results with each other and create one final file. Again, any discrepancies will be resolved after discussion with a third researcher. We will contact authors for any relevant missing data.

In case overlapping populations are analysed in multiple studies, we will include according to the following hierarchy the study that (1) has the lowest risk of bias (see risk-of-bias assessment below), (2) evaluates the most comprehensive policy, or (3) incorporates the largest sample size. In case one study reports multiple effect estimates for overlapping populations, we will select according to the following hierarchy the one derived from (1) the most adjusted model, (2) the longest observation period, (3) the most comprehensive policy, or (4) the largest treatment group.

Data items

From eligible studies we will extract the following items: (1) the first author's name, (2) his or her affiliation, (3) publication year, (4) type of publication (journal, book, dissertation etc.), (5) access information (URL or doi), (6) study design, (7) observational period (the beginning and end dates), (8) exact places covered by the policy, (9) whether the policy covers outdoor or indoor places, (10) whether the policy covers public or (semi) private, places, (11) timing of policy, (12) institution/government that initiated the policy, (13) country/location where the policy had been implemented, (14) compliance with the policy, (15) enforcement of the policy, (16) eligibility criteria for inclusion, (17) description of control group(s), (18) population at risk, (19) number of participants/events, (20) control and treatment group size, (21) characteristics of the population and the treatment groups (e.g. age, gender, and socioeconomic status), (22) data source(s) used for the study, (23) definition(s) of outcome measure(s), (24) controlled confounders (if applicable), (25) applied statistical techniques to draw inference, (26) number of clusters (if applicable), (27) cluster size (if applicable), (28) whether the results were adjusted for clustering (if clustered study), (29) whether the intra-class correlation coefficient is reported and what it is (if clustered study), (30) the number of drop-outs/missing values, (31) techniques for handling missing values, (32) pre-intervention population at risk(n) / events(n) / rates (%) of outcome variable(s), (33) post-intervention population at risk(n) / events(n) / rates (%) of outcome variable(s), (34) association between smoke-free policy and outcome(s) (coefficients, confidence intervals, and p-values), (35) any unintended effects (e.g. TSE displacement), (36) proportion of children exposed to TSE in the location covered by the smoke-free policy, before and after the implementation of the policy, (37) risk ratios of respiratory disease for different levels of TSE according to specific locations covered by the smoke-free policy, (38) bias assessment (see section *Risk of bias assessment*), (39) elements supporting causal inference (see section *Elements supporting causal inference*), (40) any conflict of interest reported by the authors, and (41) the funding source(s).

Outcomes and prioritisation

As previously detailed, we will identify studies evaluating the effects of smoke-free policies on children's (1) TSE and (2) health outcomes. See the description of outcomes and our prioritisation in the *Eligibly criteria* section.

Risk of bias assessment

For each individual result, we will assess risk of bias using standardised assessment tools. For randomised trials, we will use the *Quality Assessment Tool for Quantitative Studies,* developed by EPOC.[23] For non-randomised studies, we will use the *Risk Of Bias In Non-randomised Studies of Interventions* (ROBINS-I) tool.[24] The ROBINS-I tool evaluates biases in the following domains: (1) bias due to confounding (Appendix Table 1), (2) bias in selection of participants into the study, (3) bias in classification of interventions, (4) bias due to deviations from intended interventions (See Appendix), (5) bias due to missing data, (6) bias in measurement of outcomes, and (7) bias in selection of the reported result. For each domain, we will rate the risk of bias on a 4-point scale ranging from critical risk of bias to low risk of bias (i.e. bias is comparable to a well-performed randomised trial). The ROBINS-I tool will be followed to give an overall estimate of the risk of bias. The risk-of-bias assessments will be conducted independently by two researchers and a third reviewer will be consulted to resolve disagreements.

Elements supporting causal inference

Based on information reported in the included studies, we will reflect on elements that may support causal inference and the robustness of the evidence using the UK Medical Research Council guidance on natural experiments.[25] First, we will report whether the effect estimates were robust when using alternative comparison groups (e.g. the use of

For peer review only - http://bmjopen.bmj.com/site/about/guidelines.xhtml

different populations living in distinct geographical areas as a comparison). Second, we will report whether a study estimated whether the smoke-free policy also had an 'effect' on neutral outcomes that were not expected to change as a consequence of the intervention (e.g. hospital admission for appendicitis). Finally, we will report from the included studies any additional information from complementing research methodologies regarding possible underlying mechanisms supporting the quantitative study findings. These assessments will be based on the quantitative and qualitative information presented in the individual study reports. We anticipate that this information cannot be uniformised across studies, and as such will be reported narratively.

Data synthesis

I. Synthetisation of data

Obtaining comparable data is essential to facilitate meta-analysis. Studies should be sufficiently similar in terms of their designs and the definitions of their measurements. We will uniformise outcome assessments before conducting data analysis. First, if continuous outcomes appear on different scales across studies we will use standardised mean differences. If continuous outcomes appear on the same scale, weighted mean differences will be used. Second, for dichotomous outcomes we will extract relative risks. If only absolute risks are presented then we will calculate relative risks. Third, we will express the effects on dichotomous outcomes using risk ratio (RR). If RR was not reported, we will contact the authors to provide it to us. If the requested data was not be obtained, we will convert odds ratio to RR using the following formula:

$$RR = \frac{OR}{(1 - PEER) + (PEER \times OR)}$$

, where $PEER$ refers to the patient-expected event rate in the control group.[26] In case $PEER$ is not available, we will approximate this measure using the overall event rate across the entire study population. We will use incidence rate ratios (IRRs) instead of RR for outcomes that could occur repeatedly with the same individual (i.e. hospitalisation). To make different TSE measures comparable, we will convert them into a binary variable with a value of 1 if the child was exposed to (detectable amounts of) TSE and 0 if unexposed to (detectable amounts of) TSE. Finally, two researchers will check independently whether the definitions of intervention, population, and outcomes are sufficiently comparable across the selected studies to allow meta-analysis.

The eligible studies are likely to be heterogeneous in various important characteristics which will be taken into account in the data synthesis. Therefore we will apply random-effects models (as opposed to fixed-effect) for data analysis of two or more studies with similar policies and outcome measure. Furthermore, it might be needed to take into account the dependency of observations, for example, if multiple effect estimates for similar policies across various regions were provided within a single study. A three-level meta-analysis will be considered if multiple estimations are extracted from the same study. The decision to perform a 2-level or 3-level meta-analysis will be based on the model performance using a one-sided log-likelihood-ratio test.[27,28] We will separately pool step changes (immediate changes) and slope changes (gradual changes) for each outcome in different models. If unspecified, the result will be considered a step change. We will conduct separate meta-analyses for various subtypes of smoke-free policies, according to the locations that they cover. Thus we will separately pool studies for (1) smoke-free policies covering indoor private places (e.g. cars), (2) smoke-free policies covering indoor semi-private places (e.g. multi-unit housing), (3) smoke-free policies covering outdoor private or semi-private spaces (e.g. shared gardens), and (4) smoke-free policies covering outdoor public places (e.g. parks, school grounds, beaches, and hospital grounds). Finally, regarding TSE we will pool separate models to assess unintended effects of the policy in places not covered by the policy.

The estimations of our meta-analysis will be displayed in forest plots. Heterogeneity will be quantified by the I^2 statistic for each pooled model.

We anticipate that most studies will have assessed the impact of novel smoke-free policies on TSE rather than on health outcomes. As the relationship between TSE and respiratory outcomes among children is well established,[29,30] we will use data from studies estimating the impact of novel smoke-free policies on child TSE to quantify the potential impact of these changes on our primary health outcomes. Thus, we will perform a health impact assessment and

For peer review only - http://bmjopen.bmj.com/site/about/guidelines.xhtml

quantify the proportion of children with respiratory disease that can be prevented by the change in TSE following smoke-free policies. First, we will extract the risk ratio of respiratory disease for those children who were exposed to TSE versus those who were not either from the studies included in our systematic review or from previous meta-analyses that quantified this association.[29 30] Preferably, this risk ratio will be retrieved for the specific locations that are covered by the smoke-free policy and for distinct respiratory diseases. Second, based on the risk ratios and the difference in the proportion of children exposed to TSE before and after the policy implementation, we will calculate two Population Attributable Fractions (PAFs). One quantifies the proportion of each outcome that can be attributed to TSE before the smoke-free policy and the other after the policy's implementation. The difference of these PAFs will give us the Potential Impact Fraction (PIF) capturing the change in the outcomes when the TSE level changes following a novel smoke-free policy's implementation.

II. Sensitivity analyses

Three sets of sensitivity analyses will be conducted. First, excluding results with a higher risk of bias might lead to different estimations. Thus, we will conduct meta-regression analyses using the overall risk of bias based on the ROBINS-I assessment to test if effect sizes differ according to the overall risk of bias. Second, the question may arise whether a less restrictive selection strategy regarding study design would lead to different results. We will check for the robustness of our results by additionally including studies applying not only methodologies considered as the state-of-the-art in the *Cochrane EPOC guidelines*, but also study designs with a larger risk of bias.[20] Third we will test the sensitivity of our results to the selection of studies when overlapping populations are analysed in multiple studies.

III. Subgroup analyses

We will conduct subgroup analyses to evaluate whether smoke-free policies have differential effects among certain subgroups. To assess whether policies introduced in high-income countries have a different effect than policies introduced in low- or middle-income countries, we will perform meta-regression based on the World Bank classification.[31] Second, previous studies have shown that smoke-free policies have a different effect according to individuals' SES.[21 32-34] We will extract information from subgroup analyses by SES indicators where possible and perform separate meta-analyses for high and low SES groups.

Meta-bias assessment

In each meta-analysis, we will assess the extent of publication bias via funnel plots when at least ten studies are available. Furthermore, we will look for unpublished studies for which a protocol was registered, and we will contact the authors of these studies to obtain their results. Subsequently, we will compare the findings of these unpublished studies with those of the published reports. Finally, we will look for published reports that followed a registered protocol. We will compare these protocols with the final papers to check whether selective reporting was present at any stage.

Confidence in cumulative estimate

We will discuss the strength of the evidence on the association between implementation of smoke-free policies covering (semi)private or/and outdoor places and the TSE and child respiratory health outcomes based on the effect sizes, the variances of the estimated effects, the estimated level of heterogeneity between studies, sensitivity analyses, subgroup analyses, risk of bias, elements supporting causal inference and publication bias.

3. ETHICS AND DISSEMINATION

Ethical approval will not be required because we will only be using aggregate-level published data from previous studies. We expect to complete the study until September 2020 and before submitting for publication we will update our search and include any additional eligible papers that may be identified. We will publish our results in a peer-reviewed international journal and report the research findings according to the PRISMA guideline.[35] Finally, we will disseminate our results to the public and policy-makers following the acceptance of our paper (e.g. actively seek media-attention and interaction with policy-makers and other relevant stakeholders).

For peer review only - http://bmjopen.bmj.com/site/about/guidelines.xhtml
For peer review only

Contributors JVB secured funding for this work. All authors contributed to the conceptualisation of the review and developed the methods. MKR, FJM, and JVB drafted the manuscript and AS, CM, WB, AB, and FJL provided feedback on manuscript drafts. JVB and WB developed the search strategy. All authors approved the manuscript.

Funding This study is funded by a project grant from the Dutch Heart Foundation, Lung Foundation Netherlands, Dutch Cancer Society, Dutch Diabetes Research Foundation, and the Netherlands Thrombosis Foundation. The Public Health Policy Evaluation Unit at Imperial College London is grateful for the support of the NIHR School of Public Health Research.

Competing interests None declared.

Patient consent for publication Not required.

Acknowledgement: We thank Lauren Westenberg, Brigit Toebes and Martine Bouman for providing input.

REFERENCES

1. Öberg M, Jaakkola MS, Woodward A, et al. Worldwide burden of disease from exposure to second-hand smoke: a retrospective analysis of data from 192 countries. *The Lancet* 2011;377(9760):139-46.
2. Faber T, Kumar A, Mackenbach JP, et al. Effect of tobacco control policies on perinatal and child health: a systematic review and meta-analysis. *The Lancet Public Health* 2017;2(9):420-37.
3. Toebes B, Gispen ME, Been JV, et al. A missing voice: the human rights of children to a tobacco-free environment. *Tobacco Control* 2018;27(1):3-5.
4. Jarvis MJ, Sims M, Gilmore A, et al. Impact of smoke-free legislation on children's exposure to secondhand smoke: cotinine data from the Health Survey for England. *Tobacco Control* 2012;18(1):749-61.
5. Akhtar PC, Currie DB, Currie CE, et al. Changes in child exposure to environmental tobacco smoke (CHETS) study after implementation of smoke-free legislation in Scotland: national cross sectional survey. *BMJ* 2007;335(7619):545-49.
6. Holliday JC, Moore GF, Moore LA. Changes in child exposure to secondhand smoke after implementation of smoke-free legislation in Wales: a repeated cross-sectional study. *BMC Public Health* 2009;9(1):430.
7. Sims M, Bauld L, Gilmore A. England's legislation on smoking in indoor public places and work-places: impact on the most exposed children. *Addiction* 2012;107(11):2009-16.
8. Nanninga S, Lhachimi SK, Bolte G. Impact of public smoking bans on children's exposure to tobacco smoke at home: a systematic review and meta-analysis. *BMC Public Health* 2018;18(1):749.
9. Hone T, Szklo AS, Filippidis FT, et al. Smoke-free legislation and neonatal and infant mortality in Brazil: longitudinal quasi-experimental study. *Tobacco Control* 2020(29):312-19.
10. Byron MJ, Cohen JE, Frattaroli S, et al. Implementing smoke-free policies in low-and middle-income countries: A brief review and research agenda. *Tobacco induced diseases* 2019;17
11. Painter K. Outdoor smoking: fair or foul? *BMJ* 2019;366:l5754.
12. Laverty AA, Hone T, Vamos EP, et al. Impact of banning smoking in cars with children on exposure to second-hand smoke: a natural experiment in England and Scotland. *Thorax* 2020
13. Faber T, Mizani MA, Sheikh A, et al. Investigating the effect of England's smoke-free private vehicle regulation on changes in tobacco smoke exposure and respiratory disease in children: a quasi-experimental study. *The Lancet Public Health* 2019;4(12):607-17.
14. Been JV, Nurmatov UB, Cox B, et al. Effect of smoke-free legislation on perinatal and child health: a systematic review and meta-analysis. *The Lancet* 2014;383(9928):1549-60.
15. World Health Organization. Report on the global tobacco epidemic: offer help to quit tobacco use: executive summary. 2019
16. Shamseer L, Moher D, Clarke M, et al. Preferred reporting items for systematic review and meta-analysis protocols (PRISMA-P) 2015: elaboration and explanation. *BMJ* 2015;4(1):1-9.
17. Higgins JP, Green S. Defining the review question and developing criteria for including studies. *Cochrane handbook for systematic reviews of interventions version* 2008;5
18. Croghan IT, Ebbert JO, Hays JT, et al. Impact of a countywide smoke-free workplace law on emergency department visits for respiratory diseases: a retrospective cohort study. *BMC Pulmonary Medicine* 2015;15(1):6.

For peer review only - http://bmjopen.bmj.com/site/about/guidelines.xhtml

19. World Health Organization. WHO report on the global tobacco epidemic, 2017: monitoring tobacco use and prevention policies. https://www.who.int/tobacco/global_report/2017/en/ (Accessed: 05.05.2019): World Health Organization 2017.
20. Higgins JP, Green S. Cochrane Handbook for Systematic Reviews of Interventions Version 6. 2018
21. Been JV, Millett C, Lee JT, et al. Smoke-free legislation and childhood hospitalisations for respiratory tract infections. *European Respiratory Journal* 2015;46(3):697-706.
22. Bramer WM, Giustini D, de Jonge GB, et al. De-duplication of database search results for systematic reviews in EndNote. *Journal of the Medical Library Association: JMLA* 2016;104(3):240.
23. (EPHPP) EPHPP. Quality Assessment Tool for Quantitative Studies. 2010
24. Sterne JA, Hernán MA, Reeves BC, et al. ROBINS-I: a tool for assessing risk of bias in non-randomised studies of interventions. *bmj* 2016;355:i4919.
25. Craig P, Cooper C, Gunnell D, et al. Using natural experiments to evaluate population health interventions: new Medical Research Council guidance. *Journal of Epidemiology and Community Health* 2012;66(12):1182-86. doi: 10.1136/jech-2011-200375
26. Cummings P. The relative merits of risk ratios and odds ratios. *Archives of Pediatrics & Adolescent Medicine* 2009;163(5):438-45.
27. Assink M, Wibbelink CJ. Fitting three-level meta-analytic models in R: A step-by-step tutorial. *The Quantitative Methods for Psychology* 2016;12(3):154-74.
28. Cheung MW-L. Modeling dependent effect sizes with three-level meta-analyses: a structural equation modeling approach. *Psychological Methods* 2014;19(2):211.
29. Vardavas CI, Hohmann C, Patelarou E. The independent role of prenatal and postnatal exposure to active and passive smoking on the development of early wheeze in children. *European respiratory …* 2016
30. Jones LL, Hashim A, McKeever T, et al. Parental and household smoking and the increased risk of bronchitis, bronchiolitis and other lower respiratory infections in infancy: systematic review and meta-analysis. *Respiratory research* 2011;12(1):5.
31. World Bank. World Bank Open Data: Access: https://data.worldbank.org/ (Downloaded: 07.09.2019), 2019.
32. Hajdu T, Hajdu G. Smoking ban and health at birth: Evidence from Hungary. *Economics & Human Biology* 2018;30:37-47.
33. Vicedo-Cabrera AM, Schindler C, Radovanovic D, et al. Benefits of smoking bans on preterm and early-term births: a natural experimental design in Switzerland. *Tobacco Control* 2016;25(2):135-41.
34. Bakolis I, Kelly R, Fecht D, et al. Protective effects of smoke-free legislation on birth outcomes in England-a regression discontinuity design. *Epidemiology (Cambridge, Mass)* 2016;27(6):810-18.
35. Liberati A, Altman DG, Tetzlaff J, et al. The PRISMA statement for reporting systematic reviews and meta-analyses of studies that evaluate health care interventions: explanation and elaboration. *PLoS Medicine* 2009;6(7)

For peer review only - http://bmjopen.bmj.com/site/about/guidelines.xhtml

Appendix:

Search terms used for Embase.com database

('smoking regulation'/exp OR 'smoking ban'/de OR (smokefree OR ((smoking OR smoke OR tobacco) NEAR/3 (regulation* OR government* OR law OR laws OR policy OR policies OR ban OR bans OR banned OR free OR restrict* OR act OR acts))):ab,ti) AND (child/exp OR pediatrics/exp OR childhood/exp OR 'child welfare'/de OR 'child development'/de OR 'child health'/de OR 'child health care'/exp OR 'child care'/exp OR 'childhood disease'/exp OR 'pediatric ward'/de OR 'pediatric hospital'/de OR (infan* OR child* OR kid OR kids OR toddler* OR boy* OR girl* OR minors OR underag* OR (under NEXT/1 (age* OR aging)) OR kindergar* OR prepubescen* OR prepubert* OR pediatric* OR paediatric* OR school* OR preschool* OR (age NEAR/3 analy*)):ab,ti) AND ('asthma'/exp OR 'wheezing'/exp OR 'respiratory tract infection'/exp OR 'coughing'/exp OR 'otitis media'/exp OR 'exposure'/de OR 'self report'/exp OR cotinine/exp OR nicotine/exp OR 'saliva analysis'/exp OR 'urinalysis'/exp OR 'electronic device'/de OR 'wearable device'/de OR 'wearable sensor'/de OR 'hair analysis'/exp OR 'skin analysis device'/de OR 'passive smoking'/de OR 'environmental exposure'/de OR 'lung function test'/exp OR 'lung volume'/exp OR (asthma* OR wheezing OR ((respirator* OR lung OR pulmonar* OR expirat*) NEAR/3 (infection* OR function* OR volume*)) OR pneumonia OR bronchiolitis* OR cough* OR (otitis NEAR/3 media) OR exposure* OR environment* OR self-report* OR parent*-report* OR carer*-report* OR nicotine OR cotinin* OR second-hand* OR third-hand* OR secondhand* OR thirdhand* OR (passive NEAR/3 smoking) OR ((urine* OR saliva* OR hair OR hand* OR skin) NEAR/3 analy*) OR urinalys* OR ((electronic OR wearable) NEAR/3 (device* OR sensor*)) OR spirometr* OR fev OR fev1 OR fvc OR (forced NEAR/3 vital NEAR/3 capacit*) OR Tiffeneau):ab,ti) NOT ([Conference Abstract]/lim OR [editorial]/lim OR [letter]/lim)

Bias assessment criteria (confounders and co-interventions)

ROBINS-I requires to pre-specify certain risk-of-bias criteria that are relevant for the given study. For assessing the risk of bias due to confounding, we consider the following potential confounders relevant: age, gender, socio-economic status (SES) and/or ethnicity, and the pre-implementation temporal trend in the given outcome variable. We will score results as having a low risk of bias due to confounding if it controls for all of these variables, moderate risk of bias if not all confounders are controlled for but the pre-implementation trend is, high risk of bias if the pre- implementation trend is not controlled for but some of the other confounders are, and critical risk of bias if none of the listed confounders are controlled for. Other potential contextual confounders specifically for the child health outcomes are air pollution, quality of the health care system, access to health care facilities, and the vaccination programme in the country. Therefore, additionally to the criteria noted above, studies will also be considered to have a critical risk of bias due to confounding if contextual confounders were unaccounted for despite these being reported to be an issue in the study.

For assessing bias due to co-interventions (i.e. part of ‘bias due to deviations from intended interventions’), the following co-interventions are considered: policies aimed at reducing ambient air pollution, introduction of relevant health-care reforms and/or public health programmes, introduction of other tobacco control policies, change in vaccination programmes, and wars/epidemics/economic crises which influenced the health of children. We will approximate the percentage of the observation units affected by any of these uncontrolled co-interventions. When this percentage is below 10% a result will be considered to have a low risk of bias, when it ranges between 10% and 20% moderate risk of bias, between 20% and 40% high risk of bias, and above 40% it will be considered serious risk of bias.

For peer review only - http://bmjopen.bmj.com/site/about/guidelines.xhtml

Appendix Table 1. Bias due to confounding and due to deviations from intended interventions

Level of bias	Bias due to confounding	Bias due to deviations from intended interventions
**Low risk of bias**	(I) The pre-implementation temporal trends in the outcomes are controlled<br>(II) Individual characteristics are controlled<br>(III) (For health outcomes): Contextual characteristics are controlled if reported to be an issue in the study	The percentage of the units affected by any of the co-interventions is below 10%
**Moderate risk of bias**	(I) The pre-implementation temporal trends in the outcomes are controlled<br>(II) Individual characteristics are not all controlled<br>(III) (For health outcome): Contextual characteristics are controlled if reported to be an issue in the study	The percentage of the units affected by any of co-interventions is between 10% and 20%
**Serious risk of bias**	(I) The pre-implementation temporal trends in the outcomes are not controlled<br>(II) Some of the individual characteristics are controlled<br>(III) (For health outcome): Contextual characteristics are controlled if reported to be an issue in the study	The percentage of the units affected by any of co-interventions is between 20% and 40%
**Critical risk of bias**	(I) The pre-implementation temporal trends in the outcomes are not controlled<br>(II) Some of individual characteristics are controlled<br>**OR**<br>(III) (FOR health outcome): Contextual characteristics are not controlled while being reported to be an issue in the study	The percentage of the units affected by any of co-interventions is above 40%
**No information**	No information on the controlled confounding variables	No information on the controlled co-interventions

For peer review only - http://bmjopen.bmj.com/site/about/guidelines.xhtml

PRISMA-P (Preferred Reporting Items for Systematic review and Meta-Analysis Protocols) 2015 checklist: recommended items to address in a systematic review protocol*

Section and topic	Item No	Checklist item	Page
**ADMINISTRATIVE INFORMATION**			
Title:			1
Identification	1a	Identify the report as a protocol of a systematic review	-
Update	1b	If the protocol is for an update of a previous systematic review, identify as such	
Registration	2	If registered, provide the name of the registry (such as PROSPERO) and registration number	2
Authors:			
Contact	3a	Provide name, institutional affiliation, e-mail address of all protocol authors; provide physical mailing address of corresponding author	1
Contributions	3b	Describe contributions of protocol authors and identify the guarantor of the review	12
Amendments	4	If the protocol represents an amendment of a previously completed or published protocol, identify as such and list changes; otherwise, state plan for documenting important protocol amendments	11
Support:			
Sources	5a	Indicate sources of financial or other support for the review	12
Sponsor	5b	Provide name for the review funder and/or sponsor	12
Role of sponsor or funder	5c	Describe roles of funder(s), sponsor(s), and/or institution(s), if any, in developing the protocol	12
**INTRODUCTION**			
Rationale	6	Describe the rationale for the review in the context of what is already known	3
Objectives	7	Provide an explicit statement of the question(s) the review will address with reference to participants, interventions, comparators, and outcomes (PICO)	3
**METHODS**			
Eligibility criteria	8	Specify the study characteristics (such as PICO, study design, setting, time frame) and report characteristics (such as years considered, language, publication status) to be used as criteria for eligibility for the review	4-8
Information sources	9	Describe all intended information sources (such as electronic databases, contact with study authors, trial registers or other grey literature sources) with planned dates of coverage	8
Search strategy	10	Present draft of search strategy to be used for at least one electronic database, including planned limits, such that it could be repeated	8
Study records:			
Data	11a	Describe the mechanism(s) that will be used to manage records and data throughout the review	8

For peer review only

management			
Selection process	11b	State the process that will be used for selecting studies (such as two independent reviewers) through each phase of the review (that is, screening, eligibility and inclusion in meta-analysis)	8
Data collection process	11c	Describe planned method of extracting data from reports (such as piloting forms, done independently, in duplicate), any processes for obtaining and confirming data from investigators	8-ö
Data items	12	List and define all variables for which data will be sought (such as PICO items, funding sources), any pre-planned data assumptions and simplifications	9
Outcomes and prioritization	13	List and define all outcomes for which data will be sought, including prioritization of main and additional outcomes, with rationale	9
Risk of bias in individual studies	14	Describe anticipated methods for assessing risk of bias of individual studies, including whether this will be done at the outcome or study level, or both; state how this information will be used in data synthesis	9
Data synthesis	15a	Describe criteria under which study data will be quantitatively synthesised	10-11
	15b	If data are appropriate for quantitative synthesis, describe planned summary measures, methods of handling data and methods of combining data from studies, including any planned exploration of consistency (such as I^2, Kendall's τ)	10-11
	15c	Describe any proposed additional analyses (such as sensitivity or subgroup analyses, meta-regression)	11
	15d	If quantitative synthesis is not appropriate, describe the type of summary planned	
Meta-bias(es)	16	Specify any planned assessment of meta-bias(es) (such as publication bias across studies, selective reporting within studies)	11
Confidence in cumulative evidence	17	Describe how the strength of the body of evidence will be assessed (such as GRADE)	11

*** It is strongly recommended that this checklist be read in conjunction with the PRISMA-P Explanation and Elaboration (cite when available) for important clarification on the items. Amendments to a review protocol should be tracked and dated.** **The copyright for PRISMA-P (including checklist) is held by the PRISMA-P Group and is distributed under a Creative Commons Attribution Licence 4.0.**

From: Shamseer L, Moher D, Clarke M, Ghersi D, Liberati A, Petticrew M, Shekelle P, Stewart L, PRISMA-P Group. Preferred reporting items for systematic review and meta-analysis protocols (PRISMA-P) 2015: elaboration and explanation. BMJ. 2015 Jan 2;349(jan02 1):g7647.

For peer review only
